# Community peer support among individuals living with spinal cord injury

**DOI:** 10.1177/13591053231159483

**Published:** 2023-03-16

**Authors:** Joy McLeod, Christopher G. Davis

**Affiliations:** Carleton University, Canada

**Keywords:** adjustment, peer support, SCI, spinal cord injury, well-being

## Abstract

Peer support is widely assumed to help individuals with spinal cord injury (SCI) adjust, yet the evidence is mixed. We propose that peer support may be more likely to promote adjustment when informal support is lacking. To test this hypothesis, 135 individuals living with SCI receiving peer support (46.7% female; *M*_age_ = 42.36, SD = 14.83) completed an online survey assessing aspects of and satisfaction with the peer support and family/friend support that they were receiving as well as measures of adjustment. Although those reporting receiving more peer support were not any better adjusted than those reporting less, individuals who were more satisfied with the peer support they received reported better adjustment. Moreover, the relation of satisfaction with peer support with depressive symptoms was dependent on the level of family/friend support. These findings suggest that peer support is most effective among those lacking support from family and friends.

## Introduction

Spinal cord injuries can have a significant effect on one’s social relationships which has implications for both psychological and physical health. Experiencing a traumatic life event, like acquiring an SCI, makes one feel different from others, oftentimes poorly understood—or worse, rejected (Dickson et al., 2011; Veith et al., 2006). Talking with similar others (i.e. peers, other individuals living with SCI) potentially offers both practical and emotional benefits (Veith et al., 2006). Peers can address SCI-related concerns and provide empathy and acceptance in a way unmatched by other supportive relationships (Sweet et al., 2016, 2018). We propose that the effect of informal, community peer support on adjustment depends upon two factors: how peer support is measured (i.e. perceived support versus received support) and the quality of one’s social support from other informal sources (i.e. family/friends) such that the effect will be stronger for individuals with inadequate support from family and friends.

Adjustment to an SCI involves adjusting one’s goals and lifestyle to accommodate the limitations imposed by the injury. For example, acquiring an SCI may threaten one’s ability to pursue meaningful pre-injury goals (Davis and Porter, 2018). Because of all these challenges, it is perhaps not surprising that individuals with SCI are at higher risk of depression and anxiety relative to those without SCI (Migliorini et al., 2015; Post and van Leeuwen, 2012). Despite this, most people who sustain an SCI are resilient, exhibiting stable low symptoms of anxiety and depression (Bonanno et al., 2012). Therefore, it is important to determine what distinguishes those individuals who adjust well from those who do not.

One factor widely viewed as important in the adjustment process following SCI is the quality of social support available. In a systematic review of the literature on social support in the context of SCI, Müller et al. (2012) concluded that social support is consistently associated with better mental health. For example, Beedie and Kennedy (2002) found in a sample of people who had experienced a traumatic SCI that quality of social support was associated with lower levels of depression and hopelessness post-injury. Quality of social support has also been linked to subjective well-being (SWB), satisfaction with life, and quality of life among individuals living with SCI (Müller et al., 2012).

Researchers typically distinguish among the different functions of social support. Practical support includes helping people with the demands of daily life, such as giving someone a drive to an appointment. Informational support includes providing others with useful information, such as offering advice or suggestions. Finally, emotional support refers to expressions of empathy, love, trust, and care. For example, people may listen empathetically and provide reassurance and compassion. This latter type of support—allowing one the opportunity to talk about one’s SCI-related difficulties and distress in a non-judgmental manner—can facilitate adjustment but attempts to offer support may fail oftentimes by well-intentioned family and friends leaving one feeling misunderstood, unsupported, or rejected (Wortman and Lehman, 1985).

To better understand the conditions under which social support is helpful, Wortman and Lehman (1985) asked bereaved individuals to describe what had been particularly helpful since the loss of their loved one. The three most cited responses were contact with similar others, being given the opportunity to openly discuss feelings, and having others express genuine concern for them. As such it may be that talking with similar others (i.e. others with an SCI) about one’s SCI-related thoughts and issues may help when social support from other informal sources (e.g. family/friends) is constrained or unhelpful.

Using qualitative methods Veith et al. (2006) identified several aspects of peer support that differentiate it from other supportive relationships in one’s life. Peers are seen as credible since they have lived experience with the condition, and although mentors may be further along in the adjustment process, they are regarded as being “in the same boat.” Peer mentors (i.e. individuals who have lived through a similar experience and are trained to support others) understand and relate to one’s situation in a way that family and friends may not. Peers reported numerous positive outcomes which they attributed to their peer relationships, including greater appreciation for life, feeling inspired, improved self-identity, and reduced distress and fear of the unknown. Similar qualitative themes have been identified in other health contexts as well (e.g. among stroke survivors and individuals with cancer; Borregaard and Ludvigsen, 2017; Kessler et al., 2014).

Despite the encouraging qualitative findings on peer support among people experiencing major health concerns, quantitative studies have yielded mixed results. For instance, a study comparing community-residing individuals with SCI who were receiving peer support to a similar group that was not found no difference in quality of life (Sweet et al., 2018). In contrast, an earlier study by the same research team found that the extent to which peer support needs were met was positively correlated with life satisfaction (Sweet et al., 2016).

Peer support has been studied extensively among individuals living with cancer. Some studies report positive outcomes among peer support recipients while others report no differences between peer support recipients and non-recipients, and yet others report potential adverse effects of peer support (Campbell et al., 2004; Hoey et al., 2008; Mens et al., 2016; Steginga et al., 2004). For example, in a set of three studies Helgeson et al. (1999, 2000, 2001) evaluated different types of brief support interventions among women with breast cancer. They found the peer support intervention had some short-term negative effects and was not as effective as an educational intervention in improving social and physical functioning. Of relevance to the present study, the authors found an interaction such that peer support was beneficial for women who lacked support from other sources (e.g. their significant others) but detrimental (at least in the short-term) for women who had quality social support from others; the detrimental effects were no longer present at the 3 year follow up.

We propose that peer support may be particularly important when other informal sources of support from family and friends are perceived to be inadequate. When support from family and friends is satisfactory, there is likely less need for peer support, and it may not have an additional impact on the individual’s emotional well-being. Yet when other informal sources of support from family and friends are lacking or unsatisfactory, there is likely a greater need for peer support, and therefore it may have a greater impact on the individual’s well-being.

Moreover, evidence from a variety of contexts suggests that how people feel about the support they receive (i.e. *perceived* peer support) is more consequential than simply the receipt of support (Melrose et al., 2015; Uchino, 2009). Although the concepts are intrinsically related, past research indicates perceived support is more strongly (and consistently) associated with health and well-being than received support whether it is measured in terms of perceived availability of support or perceived adequacy of received support (Helgeson, 1993; Uchino, 2009). It may be that knowing that individuals are available and willing to provide support—they have your back—allows one to maintain a sense of personal agency. In contrast, an index of received support may be less strongly associated with well-being because it may include imposed or unrequested help, and may ignore the negative implications of receiving that help (e.g. feelings of dependency or disability).

### The present study

Peer support within the community is more flexible and variable (i.e. informal) than most of the peer support interventions previously reviewed. Individuals can connect with similar others through formal mentorship programs (where the peer has received training) or through informal connections with others who have not received training. Organizations may also offer drop-in programming (e.g. support groups and adaptive sports) or opportunities for advocacy and outreach. Peer support can also be accessed through online platforms, such as reddit (e.g. r/spinalcordinjuries community). In sum, peer support in the community can vary in format, services, training, and structure.

### Hypothesis 1

Although qualitative studies suggest that peer support has many benefits for the recipient of the support, peer support is likely to be most valuable for the recipient when support from family and friends is lacking or constrained. We therefore hypothesized that the association of received peer support with symptoms of depression and subjective well-being (SWB) would be stronger for individuals with little to no social support from other informal sources (family/friends) relative to those with high levels of support from family and friends.

### Hypothesis 2

We also predicted that individuals who perceived the peer support they were receiving as more satisfactory would experience fewer depressive symptoms and higher levels of SWB.

## Method

### Participants

Individuals with SCI who received peer support were recruited to participate in a survey of peer support through social media, SCI Ontario’s Community Magazine, and other Canadian and US SCI organizations. Of the 232 who began the survey, the data for 28 participants were discarded because they failed to complete at least 75% of the survey, 24 participants were removed for completing the survey in less than 10 minutes, 13 participants were removed for not meeting the eligibility criteria (i.e. they indicated they had no SCI peers), and 32 participants were removed for response sets, nonsense responses, or duplicate responses to one or more open-ended items. After these exclusions, we retained a sample of 135 community-living individuals (72 men, 63 women; *M*_age_ = 42.36, SD = 14.83) who have acquired an SCI. Roughly half of the sample (51.9%) had acquired their injury 5+ years ago, 43.7% had acquired their injury less than 5 years ago, and 4.4% did not disclose when they acquired their injury. See supplemental data (Table S1) for detailed sample characteristics.

We aimed a priori to recruit a sample of 150 individuals, which would yield power of .80 to detect a significant interaction (see Supplemental Material for details).

### Procedure

Potential participants expressing interest who met criteria (i.e. those who had sustained an SCI, participated in peer support, and were at least 18 years old) were emailed a link to the online survey. Participants were also given the option of completing the survey in an interview format by phone (*n* = 1). After providing informed consent, participants were asked a series of demographic questions and basic questions about their injury. They then completed surveys assessing social support from family and friends, peer support, depressive symptoms, and SWB. As an incentive, those who participated were entered into a draw for one of three gift cards. The survey took approximately 15 minutes to complete. The study was conducted between December 2020 and April 2021. Ethics approval was obtained from the authors’ university research ethics committee.

### Materials

#### Demographics

Participants were asked their gender, geographic location, age, level of education, and current living situation. They were also asked about their SCI, specifically injury etiology, time since injury (TSI), and injury severity (i.e. injury level and classification).

#### Social support from family and friends

Social support from family and friends was assessed with an adapted version of the Multidimensional Scale of Perceived Social Support (MSPSS; Zimet et al., 1988). The MSPSS assesses an individual’s perceptions of social support from a significant other (i.e. “special person”), family, and friends. The scale was modified from the original 12 items to 9, and to distinguish family/friend/special friend support from peer support, we added “who does not have an SCI” to the special person items and “non-SCI” to the family and friend items (e.g. “You get the emotional help and support you need from your family and/or non-SCI friends”). Each statement was rated on a 7-point scale ranging from *strongly disagree* to *strongly agree*. Confirmatory factor analysis indicated that although all special person items loaded on one factor and all friends and family support items loaded on a second factor, the two factors were very highly correlated (*r* = 0.87), suggesting only one factor. Therefore, scores were combined into a single mean. The MSPSS demonstrated excellent reliability in the present study, α = 0.92.

#### Peer support

Received peer support for persons with SCI was measured with the Spinal Cord Injury Peer Support Inventory (SCI-PSI), which was created for this study using the Prostate Cancer Peer Support Inventory as a guide (Steginga et al., 2004). Ten of the original 14 items were adapted to be relevant to individuals living with SCI.

Prior to deployment in this study, the draft version of the SCI-PSI was evaluated by an experienced peer mentor with SCI Ontario and her recommendations were adopted. The final SCI-PSI has 15 items, each rated on a 5-point Likert scale ranging from 1 (*strongly disagree*) to 5 (*strongly agree*; see Supplemental Data, Table S2). The survey was pilot tested with four persons living with SCI.

The SCI-PSI was designed to have three subscales which were driven by qualitative enquires (Gifre et al., 2014; Haas et al., 2012; Veith et al., 2006); they measure the extent to which individuals report that they receive practical, emotional, and identity-changing support (e.g. “my SCI peers have helped me regain a sense of purpose in my life”) from peers. Confirmatory factor analysis indicated that although each item loaded strongly on its factor, the three factors were very highly correlated (*r* = 0.91–0.93), suggesting only one factor. Therefore, it was combined into a single mean score. The SCI-PSI demonstrated excellent reliability in the present study, α = 0.95.

Prior to completing the SCI-PSI, participants were asked questions about the nature of the peer support they receive. They were asked how many peers they are in contact with, how often they are in contact with them, whether they interact with them on an individual basis or in a group format (or both), and whether their peers have received training (see Table S3 for details). Participants were also asked how satisfied they are with the peer support they receive; satisfaction was rated on a 5-point scale ranging from *very dissatisfied* to *very satisfied*. They were also asked if any of these factors had changed (and how) due to the COVID-19 pandemic. Finally, they were asked to report what aspects of peer support are most valuable to them and what aspects are least valuable or impact them negatively.

#### Depressive symptoms

The 10-item Center for Epidemiologic Studies Depression Scale (CESD-10; Andresen et al., 1994) was used to assess depressive symptoms. Participants were asked how often, in the past week, they have felt or behaved in certain ways, such as “I felt depressed.” Responses were rated on a scale ranging from 0 (*rarely or none of the time: less than 1* *day*) to 3 (*all of the time: 5–7* *days*). The CESD-10 is a widely used and well-validated self-report measure of depression (Björgvinsson et al., 2013). The CESD-10 demonstrated very good reliability in the present study, α = 0.88.

#### Subjective well-being

Diener (1984) proposed that SWB is comprised of three major components: the presence of positive affect, the relative absence of negative affect, and the sense that one’s life is satisfying. Given the tripartite structure of SWB, a composite score was created using the Positive Affect (PA) subscale, the Negative Affect (NA) subscale, and the Satisfaction with Life Scale (SWLS; Diener et al., 1985).

PA and NA were assessed using a slightly revised version of the PANAS (Watson et al., 1988). Participants were asked how often in the past few weeks they had felt 11 positive and 12 negative emotions. The PA subscale was revised to include the low arousal, positive valence mood state “calm,” and the adjective “strong” was replaced with “confident” to avoid the misinterpretation of physical strength. The NA subscale was modified slightly to include “frustrated” and “annoyed” —two negative emotions relevant to people with SCI due to the challenges they face (Davis and Novoa, 2013). Each of the affective states were rated on a scale ranging from 1 (*not at all*) to 5 (*very often).* Finally, the SWLS is a 5-item scale which was used to assess how satisfied participants are with their life. Participants were asked to what extent they agree or disagree with each statement (e.g. “If I could live my life over, I would change almost nothing.”) on a 7-point Likert scale, ranging from 1 (*strongly disagree)* to 5 (*strongly agree)*.

Each component was then transformed to a Proportion of Maximum Possible (POMP) before being averaged with the other components (Cohen et al., 1999). To calculate POMP, a participant’s score on each measure was converted to a proportion of the highest possible score, with 0 representing the lowest possible score and 1.0 representing the highest possible score. A SWB score for each participant was then obtained by averaging his or her POMP score for satisfaction with life, positive affect, and reverse-scored negative affect.

### Analysis strategy

A series of correlations were conducted to determine the relation between peer support and indicators of healthy adjustment (i.e. depressive symptoms and SWB) as well as support from family and friends. Next, we conducted a hierarchical multiple linear regression to assess the extent to which the different support variables (i.e. support family/friends and peer support) uniquely predicted adjustment. Finally, we tested an interaction model to assess whether the effect of peer support on adjustment following SCI depended upon the level of support from family and friends.

## Results

### Preliminary analysis

T-tests and ANOVAs were conducted to determine whether demographic variables were significantly associated with any variables of interest, specifically family/friend support, peer support, satisfaction with peer support, depressive symptoms and SWB. Notably, individuals who had sustained their injury within the last 5 years had significantly more depressive symptoms (*M* = 12.26, SD = 0.69) than those who had sustained their injury more than 5 years ago (*M* = 9.70, SD = 0.67), *t*(126) = −2.13, *p* = 0.035.

Over half of the sample (59.3%) reported levels of depression that met or exceeded the threshold for major depression (⩾10; Andresen et al., 1994). In terms of SWB, the sample average was 55.6% of maximum possible (SD = 17.6). Whereas a significant portion of the sample was not well-adjusted according to these criteria, ratings of support from family and friends tended to be highly positive (*M* = 4.15; SD = 0.79) as were ratings of satisfaction with peer support (*M* = 3.95; SD = 0.95). The mean for peer support was close to the mid-point of the scale (*M* = 3.67; SD = 0.94).

Table 1 summarizes correlations between the support variables and adjustment variables of interest. Perceived support from family and friends was significantly associated with the variables of interest, specifically symptoms of depression and SWB (0.29 < |*r|* < 0.35, *p*s < 0.001). Peer support was significantly associated with support from family and friends (*r* = 0.21, *p* = 0.013), however it was not significantly associated with any other variables of interest, particularly symptoms of depression or SWB (−0.03 < *r* < 0.09, *p*s > 0.314).

**Table 1.** Correlations between dependent and independent variables.

Variable (range)	*M* (SD)	1	2	3	4
1. Perceived social support (MSPSS; 1–5)	4.15 (0.79)	–			
2. Received peer support (1–5)	3.67 (0.94)	.21*	–		
3. Satisfaction with peer support (1–5)	3.95 (0.95)	.41**	.21*	–	
4. Depressive symptoms (0–30)	10.95 (6.77)	−.35**	−.03	−.32**	–
5. SWB (0–100)	55.59 (17.55)	.29**	.09	.37**	−.79**

*N* = 135.

* *p* < 0.05. **p < 0.01 (two-tailed).

#### Peer Support

On average the sample was satisfied with the support they receive from their peers (*M* = 3.95; SD = 0.95) and rated having other people in their condition available to connect with as important to very important (*M* = 4.24, SD = 0.82). To illustrate, one participant stated,

The connection I have with my peers is difficult to put into words. There is a sense of unity we share in the fact that we know exactly what each of us is going through or experiencing, mostly as it relates to SCI-related issues (i.e., struggles with bladder and bowel management, inaccessibility, housing, employment, dating. . . etc). What I find most satisfying about the peer support I receive is that I never have to explain myself. My peers get it, they get me.

### Main analysis

#### Hypothesis 1

To test the hypothesis that the association of peer support with adjustment (i.e. symptoms of depression and well-being) would be stronger for individuals with little to no support from other informal sources (family/friends) than for those with greater support, we conducted a moderated regression analyses such that each of these dependent variables was regressed on level of peer support, level of support from friends and family, and their multiplicative interaction (after mean-centering). These analyses also controlled for TSI (time since injury) when it covaried with the dependent variable (i.e. symptoms of depression). The first regression analysis indicated that support from family and friends (*b* = −2.65, *p* < 0.001), and TSI (*b* = −2.31, *p* = 0.046) significantly predicted symptoms of depression such that more family/friend support and injuries sustained more than 5 years ago were associated with fewer symptoms of depression. The effect of peer support was not significant (*b* = 0.07, *p* = 0.914). The interaction of support from family and friends by peer support yielded a marginally significant effect (*b* = 1.42, *p* = 0.071; see Table 2, panel A).

**Table 2.** Regression of dependent variables on received peer support, satisfaction with peer support, support from family and friends, and their interaction.

A)	Dependent variable: symptoms of depression				
Predictor	*b*	SE	*t*(*df* = 120)	*p*-Value	95% CI for *b*
Constant	10.83	0.58	18.68	<0.001	9.685, 11.980
Peer support (PS)	.07	0.65	0.11	0.914	−1.207, 1.346
Family and friend support (FS)	−2.65	0.74	−3.56	<0.001	−4.118, −1.173
Time since injury	−2.31	1.15	−2.02	0.046	−4.580, −0.043
PS × FS	1.42	0.78	1.82	0.071	−0.124, 2.968
B)	Dependent variable: SWB				
Predictor	*b*	SE	*t*(df = 118)	*p*	95% CI for *b*
Constant	55.97	1.52	36.79	<0.001	52.957, 58.979
Peer support (PS)	1.05	1.69	.62	0.536	−2.292, 4.382
Family and friend support (FS)	5.80	1.97	2.95	0.004	1.902, 9.694
PS × FS	−2.34	2.07	−1.13	0.261	−6.432, 1.756
C)	Dependent variable: symptoms of depression				
Predictor	*b*	SE	*t*(df = 120)	*p*	95% CI for *b*
Constant	10.338	0.59	17.66	<0.001	9.180, 11.497
Satisfaction	−1.40	0.65	−2.17	0.032	−2.680, −0.123
Family and friend support (FS)	−2.03	0.75	−2.71	0.008	−3.521, −0.546
Time since injury	−2.50	1.09	−2.28	0.024	−4.659, −0.331
Satisfaction × FS	2.13	0.74	2.88	0.005	0.670, 3.598

*Ns* = 124–127. Peer support and family/friend support are mean centered. Simple effect analyses for Level of Satisfaction with Peer Support by Family Support: When Family Support is low (−1 SD): *b*_0_ = 11.965, *b*_1_ = −3.108, SE = 0.79, *t* = −3.95, *p* = 0.0001.

When Family Support is high (+1 SD): *b*_0_ = 8.711, *b*_1_ = 0.306, SE = 0.96, *t* = 0.32, *p* = 0.749.

Next, we assessed the extent to which peer and friend/family support predicted SWB. The first regression analysis revealed that support from family and friends (*b* = 5.80, *p* = 0.004) significantly predicted SWB such that more support from family/friends was associated with higher levels of SWB. The effect of peer support was not significant (*b* = 1.05, *p* = 0.536). The addition of the two-way interaction in Step 2 (frd/family × peer support) to the prediction of SWB was not significant (*b* = −2.34, *p* = 0.261; see Table 2, panel B).^1^

#### Hypothesis 2

We also hypothesized that individuals who were satisfied with the support they receive from their peers would be better adjusted than those less satisfied. Accordingly, satisfaction with the peer support one receives was significantly associated with fewer symptoms of depression (*r* = −0.32, *p* < 0.001) and higher SWB (*r* = 0.37, *p* < 0.001). Due to the moderate correlation between satisfaction with peer support and each of the indicators of adjustment, we conducted exploratory multiple regression analyses to assess the extent to which satisfaction and family/friend support uniquely predicted adjustment (i.e. symptoms of depression and SWB).

##### Symptoms of depression

**Figure 1.**
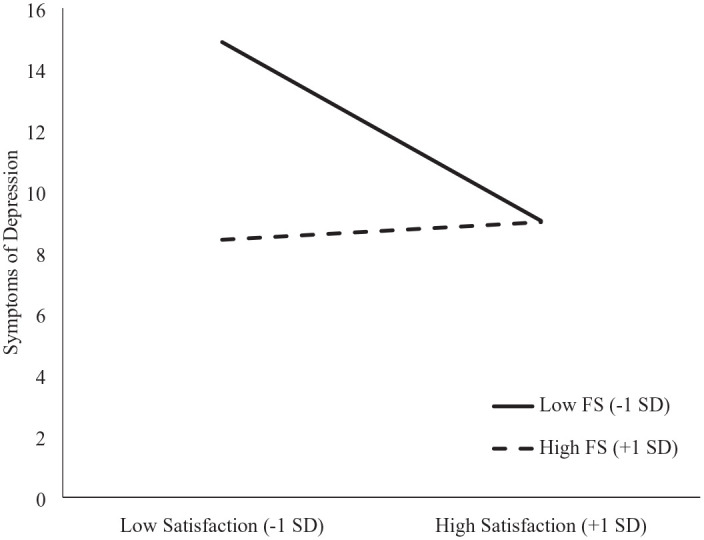
The moderating role of support from family and friends (FS) on the relation between satisfaction with peer support and symptoms of depression. This figure demonstrates the extent to which satisfaction with one’s peer support was associated with symptoms of depression as a function of support from one’s family and friends. The association of satisfaction with depressive symptoms was negative when friend and family support was low (−1 SD) and essentially zero when friend and family support was high (+1 SD). FS: family support.

The first regression analysis indicated that support from family and friends (*b* = −2.03, *p* = 0.008) and satisfaction with peer support (*b* = −1.40, *p* = 0.032) significantly predicted symptoms of depression such that more family/friend support and higher satisfaction with the peer support one receives were associated with fewer symptoms of depression. These analyses controlled for TSI, which was negatively associated with symptoms (*b* = −2.50, *p* = 0.024, such that those whose injuries were sustained more than 5 years prior were less depressed than those whose injuries were sustained more recently). The interaction of family/friend support by peer support yielded a significant effect (*b* = 2.13, *p* = 0.005; see Table 2, panel C). Simple effect analyses indicated that the effect of satisfaction with peer support was negative (*b* = −3.11, *p* = 0.0001) when family support was low (−1 SD) and essentially zero when family support was high (+1 SD; *b* = 0.31, *p* = 0.749; see Figure 1).

##### SWB

Next, we evaluated the extent to which friend/family support and satisfaction with peer support predicted SWB. Individuals with greater support from family and friends scored marginally higher on SWB (*b* = 3.87, *p* = 0.052). The effect of satisfaction with peer support was significant (*b* = 5.11, *p* = 0.004). The addition of the two-way interaction (frd/family × satisfaction with peer support) to the prediction of SWB was not significant (*b* = −3.27, *p* = 0.100).^1^

## Discussion

Despite moderately high levels of depressive symptoms and fairly low levels of SWB, participants in this study had very favorable attitudes toward peer support. By and large, they were quite satisfied with the support that they received from their peers—even over the course of the COVID-19 pandemic. Most participants also reported very high levels of support from family and non-SCI friends. Despite valuing peer support highly, the receipt of peer support was not significantly associated with symptoms of depression or SWB. In contrast, support from one’s family and non-SCI friends was consistently associated with better adjustment across all outcome measures assessed.

Whereas receipt of peer support, as measured by the SCI-PSI, did not predict adjustment, supplemental exploratory analyses indicated that satisfaction with peer support did predict both adjustment variables (i.e. symptoms of depression and SWB). The interaction of satisfaction with peer support by family/friend support in predicting depression was significant, such that the impact of satisfaction with peer support depended on the level of support from one’s family and friends. When support from family and friends was high, the association between satisfaction with peer support and depressive symptoms was essentially zero and when support from family and friends was low, the association between satisfaction and symptoms of depression was negative (i.e. those more satisfied with peer support reported being less depressed than those less satisfied).

Although contrary to our hypotheses, the finding that receipt of peer support (as measured by the SCI-PSI) was not significantly associated with indicators of psychological adjustment—even when the level of family and friend support was accounted for—has been echoed in the peer support literature (Sweet et al., 2018). As mentioned previously, past research has demonstrated that perceived support is more strongly associated with well-being than received support (Helgeson, 1993; Uchino, 2009) with some studies finding a negative association between received support and well-being (Helgeson, 1993; Komproe et al., 1997).

People differ in their social support needs. In the present study, we hypothesized that the relation between received peer support and adjustment would be stronger for individuals with poorer quality support from family and non-SCI friends. This hypothesis was based on the notion that individuals with low quality support from family and non-SCI friends may have a greater need for support, but this may not be the case. Melrose et al. (2015) found that when they measured received support as the proportion of times support was received *when needed* as opposed to solely the number of times support was received the relation between received support and well-being was strengthened. Support that is uninvited or unsolicited may undermine the individual’s agency. Likewise, support that does not satisfy the individual’s needs may have negative implications for their well-being. Our results suggest that when friend and family support is low, peer support appears to make up the difference. Perceptions of support adequacy from peers had a significant negative association with symptoms of depression, which suggests more research is needed to better understand the interplay between support needs, support from close others, and support from one’s peers.

### Limitations and future directions

Some limitations of this study should be acknowledged. First, the data are cross-sectional, so the issue of causality remains ambiguous. It is possible that greater satisfaction with the support one receives predicts better well-being, as was suggested here, but it is also possible that people who are happier tend to perceive the support they receive as more satisfying. That said, Krause et al. (1989) demonstrated that changes in satisfaction with social support preceded changes in symptoms of depression in a community sample of older adults. Yet another study found that perceived support predicted depression severity and depression recovery at a 6-month follow-up among individuals who met the diagnostic criteria for major depression (Lara et al., 1997). More recently, Prevatt et al. (2018) examined the efficacy of a peer support intervention for women experiencing postpartum depression. They found, much like in the present study, that perceptions of peer support were very positive, and satisfaction with the peer support intervention was high. Women who participated in the intervention experienced a significant reduction in depressive symptoms at follow-up.

It is also possible that other variables could be influencing the results such as the psychosocial challenges associated with the COVID-19 pandemic. Data collection for the present study took place at a time when in-person gatherings were strongly discouraged because of the prevalence of the COVID-19 virus, especially for individuals who were immunocompromised, so all peer support programs shifted online. Despite positive evaluations of peer support, many participants indicated in response to open-ended questions that they missed in-person gatherings and adaptive group sports. Ratings of satisfaction with peer support may have been adjusted by participants to take the pandemic context into account even though the efficacy of peer support may have suffered during this time because participants’ needs for human interaction and activity went unmet. Given the range of secondary health complications that occur as a result of a spinal cord injury (which can leave individuals house- or bedbound for weeks or months on end, e.g. pressure ulcers), these results likely have generalizability and importance beyond the COVID-19 context.

### Implications

It is well-established that informal social support from family and friends is a valuable (perhaps even critical) resource for people who have experienced life changing adversities. Qualitative studies suggest that support from peers—others who have experienced the same adversities—is also valuable, as peers have unique insights and knowledge based on their lived experience with the adversity. Despite the almost universal praise for peer support, studies that set out to empirically test the effect that peer support has on adjustment more often than not show null results. The present research offers two explanations why this might be the case: (1) the assessment of peer support has tended to focus on what peers receive in terms of support, rather than how satisfied they are with that support; and (2) peer support appears to be more strongly related to adjustment when informal support from friends and family is perceived to be inadequate. When support from family and friends is perceived to be satisfactory, peer support appears to have no value-added benefit, at least in terms of adjustment. When such support is lacking, however, peer support may fill the void. This interpretation is consistent with Helgeson et al.’s (2000) observation that, among people with cancer, peer support was most valuable when family support was perceived to be deficient. Therefore, it may be advisable for organizations that provide peer mentoring services to consider the informal social support an individual has and target those with less informal support available or those who are less satisfied with the informal support they are receiving from family and friends.

## Research Data

sj-docx-1-hpq-10.1177_13591053231159483 – Supplemental material for Community peer support among individuals living with spinal cord injury Click here for additional data file. sj-docx-1-hpq-10.1177_13591053231159483 for Community peer support among individuals living with spinal cord injury by Joy McLeod and Christopher G. Davis in Journal of Health Psychology This article is distributed under the terms of the Creative Commons Attribution 4.0 License ( http://www.creativecommons.org/licenses/by/4.0/) which permits any use, reproduction and distribution of the work without further permission provided the original work is attributed as specified on the SAGE and Open Access pages ( https://us.sagepub.com/en-us/nam/open-access-at-sage).

sj-docx-5-hpq-10.1177_13591053231159483 – Supplemental material for Community peer support among individuals living with spinal cord injury Click here for additional data file. Supplemental material, sj-docx-5-hpq-10.1177_13591053231159483 for Community peer support among individuals living with spinal cord injury by Joy McLeod and Christopher G. Davis in Journal of Health Psychology

sj-sav-2-hpq-10.1177_13591053231159483 – Supplemental material for Community peer support among individuals living with spinal cord injury Click here for additional data file. sj-sav-2-hpq-10.1177_13591053231159483 for Community peer support among individuals living with spinal cord injury by Joy McLeod and Christopher G. Davis in Journal of Health Psychology This article is distributed under the terms of the Creative Commons Attribution 4.0 License ( http://www.creativecommons.org/licenses/by/4.0/) which permits any use, reproduction and distribution of the work without further permission provided the original work is attributed as specified on the SAGE and Open Access pages ( https://us.sagepub.com/en-us/nam/open-access-at-sage).

sj-sps-4-hpq-10.1177_13591053231159483 – Supplemental material for Community peer support among individuals living with spinal cord injury Click here for additional data file. sj-sps-4-hpq-10.1177_13591053231159483 for Community peer support among individuals living with spinal cord injury by Joy McLeod and Christopher G. Davis in Journal of Health Psychology This article is distributed under the terms of the Creative Commons Attribution 4.0 License ( http://www.creativecommons.org/licenses/by/4.0/) which permits any use, reproduction and distribution of the work without further permission provided the original work is attributed as specified on the SAGE and Open Access pages ( https://us.sagepub.com/en-us/nam/open-access-at-sage).

sj-spv-3-hpq-10.1177_13591053231159483 – Supplemental material for Community peer support among individuals living with spinal cord injury Click here for additional data file. sj-spv-3-hpq-10.1177_13591053231159483 for Community peer support among individuals living with spinal cord injury by Joy McLeod and Christopher G. Davis in Journal of Health Psychology This article is distributed under the terms of the Creative Commons Attribution 4.0 License ( http://www.creativecommons.org/licenses/by/4.0/) which permits any use, reproduction and distribution of the work without further permission provided the original work is attributed as specified on the SAGE and Open Access pages ( https://us.sagepub.com/en-us/nam/open-access-at-sage).

## Notes

1. We also assessed community reintegration and found similar results.
