# Supplementary material for: Community peer support among individuals living with spinal cord injury
Source: J Health Psychol. 2023 Mar 16;28(10):943–55. doi: 10.1177/13591053231159483 (PMC10467001; doi:10.1177/13591053231159483)
Supplement: sj-docx-1-hpq-10.1177_13591053231159483 – Supplemental material for Community peer support among individuals living with spinal cord injury [file sj-docx-1-hpq-10.1177_13591053231159483.docx]

**Explanatory Memo**

Community Peer Support Among Individuals Living with Spinal Cord Injury

**Files**

***Peer Support Study Dataset.sav:***

| **Variable Legend** | |
| --- | --- |
| MSPSS | Non-SCI family/friend support |
| SCIPSI | Peer support |
| satis | Satisfaction with peer support |
| CESD | Depressive symptoms |
| pomp | Subjective well-being |
| RNLI | Community reintegration |

***Peer Support Study Syntax (2022).sps:*** annotated

***Peer Support Study Output.spv:*** annotated

Software: IBM SPSS (Version 28.0 1.0)
