# Supplementary material for: Community peer support among individuals living with spinal cord injury
Source: J Health Psychol. 2023 Mar 16;28(10):943–55. doi: 10.1177/13591053231159483 (PMC10467001; doi:10.1177/13591053231159483)
Supplement: sj-docx-5-hpq-10.1177_13591053231159483 – Supplemental material for Community peer support among individuals living with spinal cord injury [file sj-docx-5-hpq-10.1177_13591053231159483.docx]

Supplemental Material

# **Table S1**

# *Sample Characteristics*

| Characteristic | Percentage |
| --- | --- |
| Gender |  |
| Female | 46.7% |
| Male | 53.3% |
| Location |  |
| USA | 69.6% |
| Canada | 23.7% |
| Europe and the UK | 4.4% |
| Other (e.g., Australia) | 2.1% |
| Living Situation |  |
| Living with spouse or partner | 45.2% |
| Living with parents | 25.2% |
| Living with children | 16.3% |
| Living alone | 21.5% |
| Other (e.g., supported living) | 5.2% |
| Level of Education |  |
| Some high school | 0.7% |
| High school diploma | 7.4% |
| Some college or university | 27.4% |
| College degree | 25.2% |
| Bachelor’s degree | 22.2% |
| Masters’ or PhD | 17.0% |
| Time Since Injury |  |
| ≤5 years | 43.7% |
| >5 years | 51.9% |
| Did not disclose | 4.4% |
| Injury Etiology |  |
| Motor vehicle accident | 42.2% |
| Sports and recreation (e.g., ATV accident, diving) | 17.8% |
| Fall | 14.8% |
| Medical reasons (e.g., surgery, tumour) | 11.9% |
| Violence | 5.2% |
| Other cause (e.g., work-related injury) | 8.1% |
| Injury Classification |  |
| Tetraplegia | 40.7% |
| Paraplegia | 44.4% |
| Ambulatory | 14.8% |

**Power Analysis**

To estimate statistical power for the interaction of social support and peer support on

depressive symptoms, we assumed (based on prior literature) that the correlation of peer support with depressive symptoms would be *r* ~ -.25 (Parra et al., 2018; Stice et al., 2004), of friend/family support with depressive symptoms would be *r* ~ -.35 (Müller et al., 2012), and of friend/family support with peer support would be *r* ~ .20 (Stice et al., 2004). We further assumed that the standardized effect of the interaction of peer support with social support on depressive symptoms would be *beta* ~ .20. With these values, we used *Mplus* to estimate *n* for power of .80. These analyses indicated that a sample of 150 would yield a significant interaction effect 80% of the time.

**References**

Müller R, Peter C, Cieza A and Geyh S (2012) The role of social support and social skills in people with spinal cord injury--a systematic review of the literature. *Spinal Cord 50*(2): 94-106.

Parra LA, Bell TS, Benibgui M, Helm JL and Hastings PD (2018) The buffering effect of peer support on the links between family rejection and psychosocial adjustment in LGB emerging adults. *Journal of Social and Personal Relationships 35*(6): 854-871.

Stice E, Ragan J and Randall P (2004) Prospective relations between social support and depression: Differential direction of effects for parent and peer support? *Journal of abnormal psychology 113*(1): 155.

# **Table S2**

# *Mean Scores for the Spinal Cord Injury Peer Support Inventory (SCI-PSI)*

| Item | *M*(*SD*) |
| --- | --- |
| Your SCI peers share their personal experience with spinal cord injury. [P]  You feel that you understand your spinal cord injury better because of your relationships with your SCI peers. [P]  Your SCI peers are an inspiration to you. [I]  You receive helpful information about spinal cord injury management from your SCI peers. [P]  You know that someone in your condition is always available when you need someone to talk to. [E]  You feel a sense of connection with your SCI peers. [E]  You feel reassured by your SCI peers about how to direct your care. [P]  You feel more in control of your life because of the support you receive from your SCI peers. [I]  You can talk about your SCI-related concerns with your SCI peers. [E]  Your SCI peers have helped you realize that your life – even though it may be different – can be meaningful. [I]  You can discuss worrying thoughts and emotions with your SCI peers. [E]  You feel reassured about your personhood by your SCI peers. [I]  You receive practical information from your SCI peers about secondary health conditions. [P]  You receive support from your SCI peers about coping with your losses. [E]  Your SCI peers have helped you regain a sense of purpose in your life. [I] | 3.80(1.37)  3.64(1.22)  3.67(1.22)  3.87(1.16)  3.56(1.37)  3.84(1.22)  3.46(1.15)  3.46(1.15)  4.12(1.19)  3.65(1.32)  3.81(1.28)  3.64(1.14)  3.84(1.15)  3.41(1.22)  3.37(1.18) |

*Note.* Scores ranged from 1 = ‘strongly disagree’, to 3 = ‘neither agree nor disagree’ to 5 = ‘strongly agree.’ P = practical support item; E = emotional support item; I = identity-changing influence item.

# **Table S3**

# *Characteristics of Peer Support*

| Characteristic | Percentage |
| --- | --- |
| Format |  |
| One-on-one | 34.8% |
| Group  Both one-on-one & group | 20.5%  44.7% |
| Peer training  Yes  No  Some have received training  Unsure | 9.9%  23.0%  41.0%  26.1% |

*Note:* Participants reported being in contact with their peer(s) 2-4 times a month, on average.
